# Case Report: Prolonged Neutropenia in Premature Monoamniotic Twins With SARS-CoV-2 Infection Acquired by Vertical Transmission

**DOI:** 10.3389/fped.2022.877954

**Published:** 2022-04-25

**Authors:** Anna S. Scholz, Stephanie Wallwiener, Johannes Pöschl, Navina Kuss

**Affiliations:** ^1^Department of Gynecology and Obstetrics, University Hospital Heidelberg, Heidelberg, Germany; ^2^Department of Neonatology, University Children’s Hospital, University Hospital Heidelberg, Heidelberg, Germany

**Keywords:** SARS-CoV-2, monoamniotic twin pregnancy, neutropenia, vertical transmision, prematurity

## Abstract

**Background:**

Vertical transmission of severe acute respiratory syndrome coronavirus 2 (SARS-CoV-2) infection is a highly debated topic in the current pandemic situation. Early neonatal SARS-CoV-2 infection is rare and generally mild. Long-term data describing symptoms after COVID-19 in premature neonates is scarce.

**Case Presentation:**

Two premature, monoamniotic neonates were born by cesarean section to a mother 5 days after onset of symptomatic COVID-19. On day three of life both neonates developed hyperthermia, respiratory distress, and hematological changes, of which neutropenia persisted for over 40 days. Nasopharyngeal swabs for SARS-CoV-2 turned positive four days after delivery although the neonates were strictly isolated. Both neonates showed nearly identical time courses of ct values.

**Conclusion:**

Our case report revealed prolonged low absolute neutrophil counts in two preterm neonates with symptomatic SARS-CoV-2 infection that is reasonably assumed to have been transmitted vertically *in utero*. After preterm delivery to a SARS-CoV-2 positive mother, testing for SARS-CoV-2 infection in neonates is crucial. Both neutropenia and lymphopenia should alert physicians to test for SARS-CoV-2 infection and also to follow the case.

## Introduction

The numbers of SARS-CoV-2 infections during pregnancy are constantly increasing, thus raising concern of mother-to-fetus transmission. Current data suggest that the risk is low for neonates born to infected women to test positive for SARS-CoV-2 (1–3). Although only rarely reported, placental infection of SARS-CoV-2 appears to be possible (4). This case study summarizes the clinical course of two premature, monoamniotic neonates in whom symptomatic SARS-CoV-2 infection developed early after delivery by elective cesarean section at 32 weeks of gestation, suggesting *in utero* vertical transmission of SARS-CoV-2.

## Case Reports

A 33-year-old woman (gravida 2, para 1) was admitted to our hospital at 29 weeks and 6 days of gestation for surveillance owing to the high-risk constellation of a monoamniotic twin pregnancy.

**FIGURE 1:**
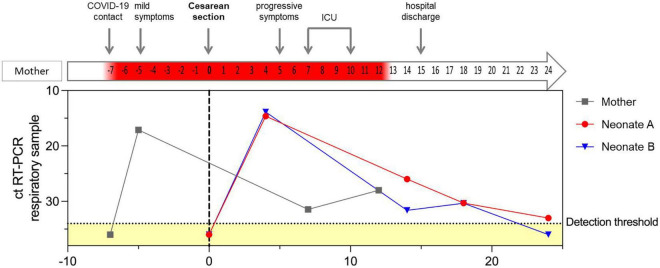
RT-PCR cycle threshold values for SARS-CoV-2 in respiratory samples of the mother and both neonates in relation to maternal clinical course. The dotted horizontal line indicates the detection threshold set at 34, distinguishing between positive and negative (marked as yellow) RT-PCR results. Negative RT-PCR results are displayed as ct values of 36. Day of cesarean section is defined as day 0, represented with a gray, vertical, dotted line. Positive RT-CPR results of the mother’s respiratory samples are highlighted in red in the arrow illustrating the maternal clinical course.

The pregnancy had gone well with regular prenatal care visits and normal ultrasound examinations. The patient had had close contact with a confirmed COVID-19 case, but tested negative for SARS-CoV-2 by RT-PCR. Two days later, however, she developed a mild cough and SARS-CoV-2 infection was confirmed by RT-PCR of her nasopharyngeal swab (Figure 1). The patient received symptomatic antitussive therapy without any need for additional oxygen supply. As aberrant patterns were intermittently detected by cardiotocography, the cesarean section was scheduled for 32 + 0 weeks of gestation. A full course of betamethasone for respiratory distress syndrome prophylaxis was administered. Elective cesarean section was then performed 5 days after onset of symptoms; the amniotic membranes were intact, there were no uterine contractions, and the patient was in full isolation under regional anesthesia.

Two female neonates were born: A weighing 1480 g and B weighing 1600 g. Cord blood analysis revealed normal pH values. At birth nasopharyngeal swabs of both neonates were tested negative for SARS-CoV-2 using RT-PCR. The neonates were placed in an incubator in an individual room of our neonatal intensive care unit (NICU) under contact and droplet isolation measures, according to current recommendations.

Shortly after birth, both neonates routinely received caffeine. Neonate A breathed spontaneously with FiO2 of 0.21. On day 3 of life, she developed fever up to 38°C and tachypnea. Therefore, treatment with ampicillin and gentamycin was started empirically for 3 days. She did not require respiratory support and fluctuating temperatures normalized within two days.

In contrast, neonate B required support *via* continuous positive airway pressure (CPAP) for respiratory distress during the first 10 h of life. Mild respiratory distress was confirmed by initial chest X-ray. Her condition remained stable until day 3 of life, when she developed secondary respiratory deterioration with dys- and tachypnea, whereupon CPAP was initiated followed by high-flow nasal cannula with respiratory improvement until day 8. Additionally, the frequency of apneas increased greatly, with repeated stimulation being needed despite additional caffeine doses. Subfebrile temperatures up to 37.6°C were observed after 78 h and stabilized spontaneously within two days.

After 91 h, SARS-CoV-2 infection was diagnosed in both neonates by RT-PCR in their nasopharyngeal swabs with low cycle threshold (ct) values and was confirmed by multiple consecutive RT-PCR tests (Figure 1). SARS-CoV-2 sequencing revealed the Delta variant in both neonates, as well as in the respiratory sample of the supposed index person of the mother (B1.617.2).

**FIGURE 2:**
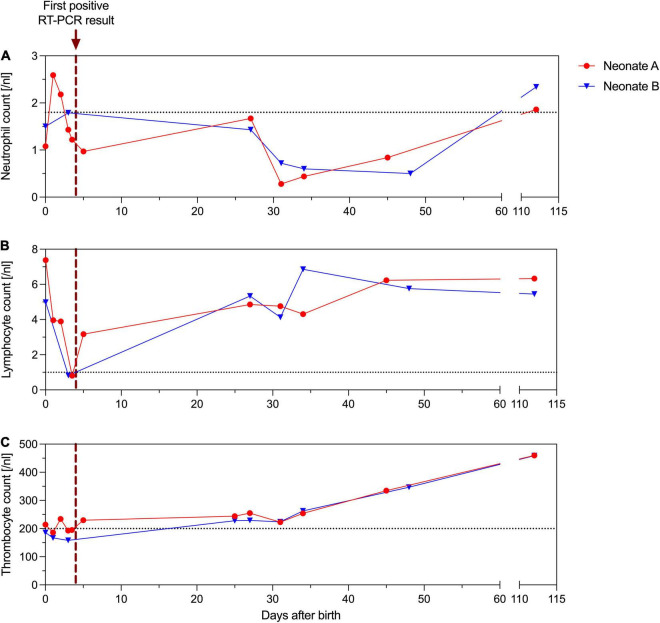
Absolute neutrophil **(A)**, lymphocyte **(B)** and thrombocyte **(C)** counts [/nl] of neonate A (red) and B (blue). The purple dotted vertical line indicates the first positive RT-PCR result of the neonates’ nasopharyngeal swab. The gray dotted horizontal line indicates lowest range of the reference interval.

Laboratory measurements of both neonates always showed normal CRP levels, whereas absolute lymphocyte counts (< 1/nl) and total white blood cell counts (< 3/nl) were low on third day of life. Absolute neutrophil levels, however, were persistently low over months (Figure 2). Both neutropenia and lymphopenia preceded the SARS-CoV-2 positive swabs. Echocardiography and ultrasound of the brain were normal in both twins. Follow-up examination after four months revealed normal development of both premature neonates.

The mother remained hospitalized for 3 days and was discharged with a mild cough. After leaving the hospital, she developed a progressive cough and elevated temperature of 38.6°C. Due to increasing respiratory insufficiency and COVID-19 pneumonia she was transferred to our intensive care unit and received 40 L/min oxygen *via* a high-flow nasal cannula, piperacillin/tazobactam, caspofungin, remdesivir, and corticosteroids. Chest CT scan revealed bilateral ground-glass opacities. High-flow oxygen therapy was gradually reduced and after 4 days on the ICU she was transferred back to a regional hospital (Figure 1).

## Discussion

We present two cases of preterm monoamniotic neonates with a symptomatic SARS-CoV-2 infection, most probably vertically transmitted *in utero via* the placenta. According to published reports, vertical transmission rates are low at about 3%, which is comparable to other congenital viral infections such as varicella or cytomegalovirus (5). COVID-19 symptoms in neonates are commonly mild or asymptomatic (6).

We are confident that we observed two actual symptomatic neonatal SARS-CoV-2 infections rather than contaminations, as a nearly identical time course for the ct values was observed in both twins. In our neonates, SARS-CoV-2 infection caused subfebrile temperatures and respiratory deterioration with severe central apneas from day 4 to 5 of life, delaying the hospital discharge of neonate B due to persistent hypopnea and apnea – persisting longer than would be expected as a result of the premature birth. Hyperthermia, respiratory distress, tachypnea, and apnea belong to the most common signs of COVID-19 in neonates (1, 6). Hematological abnormalities like low neutrophil and lymphocyte counts have already been reported (7–9). Here, these changes preceded the onset of symptoms and diagnosis of COVID-19. It is worth noting that the clinical course and the hematological changes in the two preterm neonates differed. Both preterm neonates were born with neutropenia, but only neonate A reached two average neutrophil counts before their first positive swab test, while neonate B did not reach average neutrophil counts for several months. In line with the hematological differences, the status of neonate B was inferior to that of neonate A and may reflect that their immunological response was slightly different at the beginning. Premature neonates and especially those with a disturbed immunological system at birth are naturally at high risk of respiratory illness and infections. Although neonatal SARS-CoV-2 infections are rare, newborns and especially preterm neonates require cautious observation, because they are more likely to suffer from longer intensive care and respiratory support after SARS-CoV-2 infection (1, 10). A novelty of our case is the prolonged period of low absolute neutrophil counts for several months after birth. The delayed recovery of the neutrophils may represent the unresolved inflammation in long COVID in premature neonates (11). Ryan et al. recently demonstrated an ongoing immune dysregulation by profiling the immune cell populations in adults recovering from COVID-19 (12). Only little is known yet about immunological perturbations after early SARS-CoV-2 infection in premature infants.

Unlike other case reports describing vertical transmissions of SARS-CoV-2 (13), a unique feature of our case is the monoamniotic pregnancy, which offers a case-control-like situation with nearly identical courses of infection in both twins and suggests that exposure to SARS-CoV-2 took place at an identical time point under identical conditions – like *in utero*, sharing the identical placenta. Here, intraoperative and postnatal transmission appears very unlikely. The mother continuously wore a FFP2 mask, was not intubated, and had no contact with the neonates in the timeframe between birth and onset of symptoms, making droplet and aerosol contamination very unlikely. Postnatal infection seems nearly impossible considering the extensive quarantine measures and strict hygienic rules in our NICU. All staff members in contact with the neonates were asymptomatic during and after the delivery. In theory, *in utero* transmission of SARS-CoV-2 requires viremia and placental cell infection. Several reports of placental chronic intervillositis in SARS-CoV-2-infected women have been published (4, 14). Demirjian et al. described a comparable timing and course of vertical transmission with early neonatal SARS-CoV-2 infection with an initial negative test on the day of cesarean section that turned positive on the third day of life with proven maternal viremia on the day of delivery (13). Considering the short time interval between the onset of COVID-19 symptoms and delivery in our neonates, one may suggest that viremia was present in both mother and twins at the time of cesarean section. We speculate that the delayed detection of SARS-CoV-2 in respiratory samples can be attributed to the lack of viral colonization of respiratory mucosa *in utero*, which in turn is presumably due to the lack of breathing activity before delivery. That might suggest, too, that there was no viral contamination in the amniotic fluid. Our report has some limitations. First, this report is limited to a single case of monoamniotic twins. Second, blood samples were not analyzed for RT-PCR or antibody levels and no specific PCR testing of amniotic fluid, placenta, or umbilical cord was performed to definitively verify vertical transmission.

SARS-CoV-2 infection is associated with high risk of premature delivery (10, 15). This increased risk can be explained by either a critical or worsening condition of the infected mother or obstetric reasons like preeclampsia. Furthermore, SARS-CoV-2 infections were associated with higher rates of premature rupture of the membranes (15). The risk of fetal infections generally depends on gestational age at exposure. Placental SARS-CoV-2 infection requires the co-expression of the angiotensin-converting enzyme 2 (ACE-2) receptor and transmembrane protease serine 2 (TMPRSS2), which increases with advancing gestational age and may be directly linked to placenta susceptibility to SARS-CoV-2 infection (16). Especially if maternal infection occurs shortly before birth in late second or early third trimester, the immune system of the premature fetus may not be able to cope with the infection to the same extent compared to a fetus at term.

In conclusion, this case report provides deeper insights into the clinical course of vertically transmitted SARS-CoV-2 infection in premature infants. Early hematological changes should alert physicians to test for SARS-CoV-2 infection. These findings should contribute to better prevention and care among preterm neonates born to pregnant women with acute SARS-CoV-2 infection.

## Data Availability Statement

The original contributions presented in the study are included in the article/supplementary material, further inquiries can be directed to the corresponding author.

## Ethics Statement

Ethical review and approval was not required for the study on human participants in accordance with the local legislation and institutional requirements. Written informed consent to participate in this study was provided by the participants’ legal guardian/next of kin.

## Author Contributions

AS and NK wrote the first draft of the manuscript. All authors contributed to manuscript revision, read, and approved the submitted version.

## Conflict of Interest

The authors declare that the research was conducted in the absence of any commercial or financial relationships that could be construed as a potential conflict of interest.

## Publisher’s Note

All claims expressed in this article are solely those of the authors and do not necessarily represent those of their affiliated organizations, or those of the publisher, the editors and the reviewers. Any product that may be evaluated in this article, or claim that may be made by its manufacturer, is not guaranteed or endorsed by the publisher.
